# Travelling between Two Worlds: Complement as a Gatekeeper for an Expanded Host Range of Lyme Disease Spirochetes

**DOI:** 10.3390/vetsci3020012

**Published:** 2016-06-14

**Authors:** Peter Kraiczy

**Affiliations:** Institute of Medical Microbiology and Infection Control, University Hospital of Frankfurt, Paul-Ehrlich-Str. 40, D-60596 Frankfurt, Germany; Kraiczy@em.uni-frankfurt.de; Tel.: +49-69-6301-7165

**Keywords:** *Borrelia*, spirochetes, Lyme disease, vertebrates, animal host, human, complement, immune evasion, innate immunity

## Abstract

Evading innate immunity is a prerequisite for pathogenic microorganisms in order to survive in their respective hosts. Concerning Lyme disease spirochetes belonging to the *Borrelia* (*B.*) *burgdorferi* sensu lato group, a broad range of diverse vertebrates serve as reservoir or even as incidental hosts, including humans. The capability to infect multiple hosts implies that spirochetes have developed sophisticated means to counter the destructive effects of complement of humans and various animals. While the means by which spirochetes overcome the hosts immune defense are far from being completely understood, there is a growing body of evidence suggesting that binding of the key regulator of the alternative pathway, Factor H, plays a pivotal role for immune evasion and that Factor H is an important determinant of host specificity. This review covers (i) the contribution of complement in host-specificity and transmissibility of Lyme disease spirochetes; (ii) the involvement of borrelial-derived determinants to host specificity; (iii) the interplay of human and animal Factor H with complement-acquiring surface proteins of diverse borrelial species; and (iv) the potential role of additional animal complement proteins in the immune evasion of spirochetes.

## 1. Introduction

A plethora of human and animal pathogens including bacteria (e.g., *Streptococcus pyogenes*, *S. pneumoniae*, *Staphylococcus aureus*, *S. suis*, *Neisseria meningitides*, *Yersinia enterocolitica*, *Leptospira interrogans*, *Treponema denticola*, diverse *Borrelia* spp.), fungi (e.g., *Candida albicans*), and parasites (e.g., *Plasmodium falciparum*) have developed sophisticated strategies to counteract complement as a means of evading a host´s innate immunity [1,2,3]. A central immune evasion strategy utilized by many pathogenic microorganisms involves the recruitment of the fluid phase complement regulator Factor H to the microbial surface to deregulate or inhibit the activation of the alternative complement pathway (see below). Many Factor H-binding proteins have been identified and characterized (e.g., the M protein of group A *streptococci* [4], PspC of *pneumococci* [5], PorB of *N. meningitides* [6], PorA1 of *N. gonorrhoeae* [7], YadA of *Y. enterocolitica* [8], LfHA of L. interrogans [9], FhbB of *T.* denticola [10], Complement regulator-acquiring surface proteins (CRASP) of *Borrelia* [11], and Pra1 of *C. albicans* [12]). Factor H consists of 20 so-called complement control protein (CCP) domains. Domains CCP6–8 at the N-terminus and CCP19–20 at the C-terminus are used for host cell recognition, whereas domains CCP1–4 are responsible for cofactor and decay accelerating activity. Microbial Factor H-binding proteins preferentially bind to CCP6–8 and CCP19–20 domains, thus, highlighting the role of these structures as a “hot spot of interaction” between human pathogens and Factor H [13]. 

## 2. The Complement System

**Figure 1:**
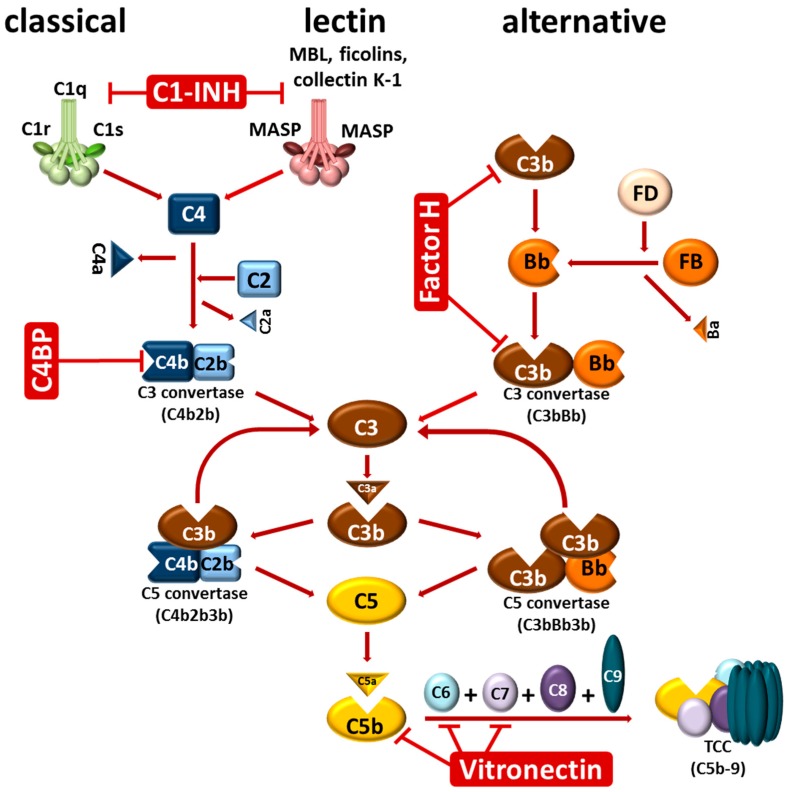
Activation of the complement system. Complement is activated in a sequential manner by three pathways: the classical, lectin, and alternative pathway. The classical pathway is initiated by binding of the C1 complex consisting of one molecule of C1q, two molecules of C1r, and two molecules of C1s to immunoglobulins (IgM, IgG) bound to their corresponding antigens. Activated C1s cleaves C4 which, upon covalent binding to the target surface, cleaves C2 leading to the formation of the C3 convertase C4b2b of the classical pathway. Activation of the lectin pathway is triggered by binding of mannan-binding lectin (MBL), ficolins (H-ficolin, L-ficolin, M-ficolin) or collectin K-1 associated with MBL-associated serine proteases MASPs to a variety of carbohydrates of microbial origin. MASP-1 and MASP-2 (MASP) are able to cleave C4 and then C2 to form the same C3 convertase. The alternative pathway is initiated by covalent binding of C3b molecules to foreign particles (opsonization). Surface-bound C3b molecules recruit Factor B (FB) leading to the formation of a C3bB complex. Following cleavage of FB by Factor D (FD), the C3 convertase (C3bBb) of the alternative pathway is generated. This enzyme cleaves C3 molecules into the small C3a fragment (anaphylatoxin) and C3b which covalently binds in close vicinity of the newly formed C3 convertases, thereby resulting in a strong amplification loop that generates an increasing number of highly reactive C3b molecules. Binding of C3b to C4b and C3b within the C3 convertase of the classical and alternative pathway leads to the formation of the C5 convertase (C4b2b3b and C3bBb3b). Binding of the additional C3b molecule changes the substrate specificity of these enzymes towards C5 which subsequently is cleaved into C5b and the most potent anaphylatoxin, C5a. Generation of C5b initiates the activation of the terminal pathway by sequential binding of C6, C7, C8, and C9 to C5b. Upon binding of multiple C9 molecules (C9 polymerization) the pore-forming terminal complement complex (TCC) integrates into the membrane and leads to lysis of susceptible cells. The soluble regulators that control activation at the level of complement initiation (C1-INH) act as cofactors for FI-mediated inactivation of C3b (Factor H) or C4b (C4BP), and prevent formation of the TCC and integration of the complex into the membrane (vitronectin).

The complement system as a central part of innate immunity represents a sophisticated network of more than 50 proteins, including inactive precursor molecules, fluid-phase and membrane-bound regulators, as well as distinct inhibitors [14,15,16,17,18]. As a first-line of defense, the most important effector function of this tightly-controlled surveillance system is the discrimination between “self” and “non-self”, leading to an immediate recognition and elimination of invading microorganisms, and thereby protecting the vertebrate host against bacterial infections. The human complement system is activated through three canonical routes: the classical, the lectin, and the alternative pathway, all of which converge in the generation and deposition of the central complement component C3b by the formed C3 convertases [15,17,19] (Figure 1). Deposition of large quantities of C3b on microbial surfaces is a prerequisite for opsonization and, ultimately, the elimination of invading pathogens via phagocytosis. Following generation of the C5 convertases, complement component C5 is cleaved into C5a and C5b which initiates the unidirectional, sequential binding of the late components C6, C7, and C8. Finally, the surface-associated C5b-8 complex initiates the polymerization of numerous C9 molecules (*n* = 10–16) following the formation of the bacteriolytic terminal complement complex (C5b-9, TCC) resulting in lysis of the invading pathogen [17,18]. Owing to the potentially deleterious effects of the complement cascade, the complement is tightly controlled at different levels by a number of soluble and membrane-anchored regulators, thus protecting self-surfaces from excessive activation and harmful attack by activated effector molecules [19]. The main soluble regulators of the classical and lectin pathway include C1 esterase inhibitor (C1-INH) and C4b-binding protein (C4BP), while the alternative pathway is primarily regulated by Factor H and Factor H-like protein 1 (FHL-1), and the terminal pathway by vitronectin [19,20].

Despite the effectiveness and abundance of complement, zoonotic microorganisms (host-adapted or pathogenic) are able to survive for a prolonged time in immunocompetent hosts and spread hematogenously to various organs and to complement-inaccessible sites, where they may cause disseminated and chronic infections. Precisely how spirochetes belonging to the *Borrelia* (*B.*) *burgdorferi* sensu lato group, circumvent the permanent alertness and destructive effects of the host’s innate immune system, exploiting specific factors and thereby interacting in multiple ways with diverse complement components, is the scope of this review.

## 3. Contribution of Complement to Host-Specificity and Transmissibility of Lyme Disease Spirochetes

The genus *Borrelia* consists of at least two major groups of vector-borne pathogens comprising the causative agents of both Lyme disease (LD) and relapsing fever [21,22]. Among the 20 *Borrelia* species belonging to the *B. burgdorferi* sensu lato complex transmitted by ixodid ticks, *B. burgdorferi*, *B. afzelii*, *B. garinii*, *B. spielmanii*, as well as *B. bavariensis* (formerly referred to as *B. garinii* OspA serotype 4) are known to be the causative agents of human LD [23]. The pathogenicity of the remaining 15 genospecies remains uncertain mainly due to the lack of culture-confirmed cases of suspected human LD, although *B. valaisiana*, *B. lusitaniae*, as well as *B. bissettii* have either been detected in biopsies by polymerase chain reaction (PCR) or isolated from a patient with vasculitis [24,25,26]. 

In nature, the enzootic vertebrate-host transmission cycle of LD *Borrelia* involves ticks as the primary vectors, infecting a diverse group of both wild and domestic vertebrate hosts, including rodents, small mammals, dogs, goats, cattle, sheep, horses, deer, reptiles, and avian hosts [27,28,29,30,31,32,33,34,35,36,37,38,39,40]. While competent reservoir hosts are often persistently infected by spirochetes, incompetent hosts, including humans, are only susceptible to incidental borrelial infection. Notably, a reservoir host may be competent for a specific genospecies but incompetent for another, indicating that the classification of a particular vertebrate host as a “competent reservoir” should be used with caution (Table 1). It is also well-known that distinct hosts differ in their attractiveness to host-seeking ticks and in their susceptibility to different borrelial species (e.g., *B. garinii* preferentially parasitizes birds but does not infect rodents, while *B. lusitaniae* has been predominately isolated from lizards) [41,42,43,44,45]. Concerning differences in reservoir competence, there is an obvious correlation between the adaptation of spirochetes to perpetuate in distinct vertebrate hosts and their ability to counter the innate immune system, in particular, the complement of the infected hosts [31,46,47,48,49,50,51] (Table 1). Hence, the serum resistance/sensitivity pattern of a particular genospecies plays a significant role for host-specificity. It also underpins the concept of a selective transmission driven by certain factors, most likely host complement, and thereby has a strong impact both on the dispersal and the global ecology of spirochetes. In addition, it has also been shown that the borreliacidal activity of the complement of a particular host appears to have a prophylactic effect by selectively eliminating spirochetes in the midgut of feeding ticks prior to transmission to the vertebrate host [52,53]. The destruction of susceptible spirochetes in the midgut of infected ticks may have a strong implication on the transmission dynamics as complement would prevent transstadial transmission of spirochetes to the next developmental stage of the tick, resulting in a pathogen-free vector.

Consistent with the current data available from *in vitro* studies with various animal sera, as well as borrelial isolates belonging to the *B. burgdorferi* sensu lato group, an obvious systematism has emerged, supporting the notion of a direct association between spirochete transmissibility and complement susceptibility. For example, *B. garinii* and *B. valaisiana*, both of which are known to be adapted to avian hosts, are highly susceptible to rodent sera; conversely, *B. afzelii*, *B. spielmanii*, *B. japonica*, *B. bissettii*, and *B. bavariensis*, all of which are adapted to rodents, are highly susceptible to avian complement but resist complement-mediated killing by rodent and human serum (Table 1) [31,54,55,56,57]. By contrast, *B. burgdorferi*, a less specialized genospecies with an expanded host range, does not display a clear species-specific transmission pattern, instead pursuing the strategy of a “generalist”, infecting a variety of vertebrate hosts. The broader spectrum of transmissibility may also explain the pattern of an intermediate serum-resistant phenotype displayed by *B. burgdorferi* when cells were challenged with different animal sera collected from avian, rodent, and ruminant hosts, respectively [31,46,48,49]. Of note, the phenotypic categorization of a borrelial strain or isolate to be serum-sensitive, intermediate serum sensitive/resistant, as well as serum-resistant is largely influenced by at least five important, mainly technical parameters (e.g., method of choice, origin of serum, sampling and storage of serum, serum concentration, and the specific borrelial strain investigated), making it somewhat difficult to compare the data published in different studies. For instance, *B. garinii* isolates belonging to different OspA serotypes were either highly sensitive or showed an intermediate serum-resistant phenotype upon incubation with the serum from the same animal host or with human serum [46]. In terms of human serum, concentrations below 20% lack any bactericidal activity against borrelial cells, which are not killed efficiently until concentrations higher than 40% are reached; thus, serum applied in lower amounts has obviously no discriminating power for the classification of borrelial genospecies [54,57]. Also, the complement activity of distinct animal sera is strongly dependent on the sampling and storage conditions, as it is well-known that, for example, mouse complement is extremely unstable, in particular the classical pathway [58]. For most animal sera studied, the complement activity has not been analyzed prior to using the sera in functional assays, probably due to the lack of validated test systems.

## 4. Species-Specific Factors Contributing to Host Specificity of Lyme Disease Spirochetes

As innate immunity, namely complement, is a prominent determinant of host specificity of *Borreliae*, various attempts have been undertaken to investigate the underlying molecular mechanisms and the critical factors involved in conferring complement resistance [59,60,61,62,63]. First and foremost, pioneering studies on complement resistance to human serum disclosed that the acquisition of complement regulators of the alternative pathway perfectly matched the serum resistance patterns of *B. burgdorferi*, *B. afzelii*, and *B. spielmanii* [59,62,64,65]. Recruitment of the key regulators of the alternative pathway, Factor H and Factor H-like protein 1 (FHL-1), by resistant spirochetes is of physiological relevance, as the *Borrelia*-bound proteins maintain their complement regulatory activity, thereby promoting the inactivation of the central complement component C3 and the termination of all downstream processes. Inhibition of the formation of the bacteriolytic TCC and its integration into the bacterial membrane efficiently protects borrelial cells from complement-mediated killing and, finally, clearance by the host’s immune system [59,62]. Conversely, serum-sensitive *B. garinii*, *B. valaisiana*, *B. andersonii*, as well as *B. lusitaniae* lack the capability to bind human Factor H and FHL-1, underpinning the pivotal role of these complement inhibitory proteins in determining serum resistance of Lyme disease *Borrelia*, with the exception of *B. bavariensis*, which employs an independent strategy to overcome complement [59,62,64,66,67,68]. Circumventing the host’s self-defense mechanisms by acquiring Factor H appears to be a general strategy used by *Borreliae*, as comparative analysis revealed that diverse borrelial species were capable of binding to Factor H from different animals, including mouse, rat, cat, dog, and sheep. Interestingly, the binding of Factor H from horse, guinea pig, and cattle have not been observed [56,69,70,71,72,73,74,75] (Table 2). Further studies have shown that *B. burgdorferi* is able to bind to Factor H from dog, goat, and cattle [74], as well as from monkey, mini pig, guinea pig, and pig, but not from duck and chicken [72]. When evaluating the published data, it has to be mentioned that in all of the performed studies, Far-Western blot analyses were performed using either denatured proteins (cell lysates or purified proteins) for capturing Factor H from different animal sera, or denatured serum proteins were immunoblotted and probed with Factor H-binding proteins (see paragraph below) as “prey” molecules [56,70,72,74,75]. While Far-Western blot analysis has been proven to be an elegant approach for detecting protein-protein interactions [76], in particular for the screening of potential, yet unknown ligands, the utilization of partially misfolded proteins as “bait” molecules might lead to false-positive results, as recently demonstrated for the interaction of human Factor H with OspE proteins [77,78]. Thus, it is prudent to confirm the data generated by this particular methodology by an additional approach using non-denatured proteins or, if possible, genetically modified cells producing the native protein on the bacterial surface. In closing, the overall Factor H binding pattern mainly resembles the pattern of serum resistance/susceptibility observed among Lyme disease spirochetes corroborating the hypothesis of a species-specific, complement-associated host selectivity.

## 5. The Role of Factor H-Binding Complement-Acquiring Surface Proteins in Evading Complement of Diverse Hosts

In the process of elucidating the molecular mechanism of serum resistance, at least five distinct borrelial proteins, collectively termed CRASPs, have been identified among *B. burgdorferi*, *B. afzelii*, and *B. spielmanii*, including CspA (CRASP-1, BBA68), CspZ (CRASP-2, BBH06), ErpP (CRASP-3, BBN38), ErpC (CRASP-4), and ErpA (CRASP-5, BBL39) which mediate binding of human complement regulators Factor H and/or FHL-1 [11,56,60,61,62,69,72,74,79,80,81,82,83,84,85,86] (Table 3). Of note, as alternative names have been used for these proteins, unfortunately, some confusion about their identities and biological functions exist in the literature.

A series of studies clearly demonstrated that purified, as well as surface-exposed, CspA of *B. burgdorferi* s.s. and *B. afzelii* bind to human Factor H [75,82,87,88,89] while contradictory data have been reported for the binding of mouse Factor H to CspA, perhaps owing to the different approaches used to assess the protein-protein interactions [75,89]. Furthermore, binding of Factor H from other competent hosts (dog, rat, rabbit, cat, sheep, horse, cow) has not been observed, leading to the conclusion that CspA might not play a prominent role in immune evasion in these potential reservoirs [75]. Considering that humans are accidental hosts for spirochetes, the specific role of CspA and its strong potential to inactivate human complement [62,88,90] remains to be elucidated, in particular as CspA is required for the colonization of spirochetes at early stages of infection (tick-to-mammal transmission) and is produced during mammal-to-tick transmission stages, but is not synthesized during established infection [91,92]. *In vitro* studies also revealed that CspA encoding genes of *B. burgdorferi* and *B. afzelii* are upregulated after 48 h following a challenge with human, mouse, and dog serum [93].

Concerning CspA orthologs belonging to the Pfam54 protein family, binding of human Factor H and FHL-1 has only been observed for CspA of *B. afzelii* and *B. spielmanii*, but not for their paralogs, as well as for the orthologs of *B. garinii*, *B. valaisiana*, *B. andersonii*, and *B. lusitaniae* [66,67,70,84]. A putative CspA ortholog of *B. bavariensis* interacting with murine Factor H has been identified by Far-Western blotting following mass spectrometry and *de novo* sequencing [70]. Furthermore, *B. bissettii* and *B. japonica* produce proteins with molecular weights (26–28 kDa) comparable to the CspA proteins of *B. burgdorferi* (25.9 kDa) and *B. afzelii* (27.5 kDa), both of which display binding capabilities for murine Factor H [70] supporting the notion of a species-specific Factor H binding pattern for CspA. Sequence polymorphisms within the interacting binding site of the different Factor H proteins or CspA orthologs are most likely responsible for the binding specificities. 

Like CspA of *B. burgdorferi*, CspZ strongly binds functionally active Factor H and FHL-1 from human serum and is able to protect borrelial cells from complement-mediated killing [86,94]. This particular molecule is highly conserved among *B. burgdorferi* isolates and is also capable of binding Factor H derived from mouse, rabbit, sheep, pig, and cow but exhibits considerably different binding properties [95] (Table 3). In contrast, CspZ orthologs identified in *B. garinii* and *B. afzelii* bound neither Factor H from these animals nor Factor H of human origin, and thus it appears highly unlikely that these molecules contribute to immune evasion through interaction with this specific complement regulator. However, CspZ orthologs may still have an impact on species-specific host-pathogen interactions by binding as yet unknown serum proteins [95]. 

Besides CspA and CspZ, additional infection-associated proteins belonging to the OspE/F-related (Erp) protein family, namely ErpA (CRASP-5, BBL39), ErpC (CRASP-4), and ErpP (CRASP-3, BBN38) of *B. burgdorferi* have been identified as potential ligands for human Factor H and the Factor H-related proteins FHR-1, FHR-2, and FHR-5 [11,60,69,71,72,74,78,80,96,97,98] along with distinct OspE orthologs of *B. afzelii*, *B. lusitaniae*, *B. japonica*, *B. spielmanii*, *B. andersonii*, *B. turdi*, and *B. tanukii* [64,66,70,85,99]. Curiously, in the absence of CspA and CspZ, *Borreliae* do not bind to Factor H, despite producing Erp proteins, but interact strongly with various FHRs, indicating that these particular molecules exhibit different binding properties *in vivo* than the respective purified proteins. Owing to the strong binding affinity of FHR-1 and FHR-2 for ErpA, ErpC, and ErpP, it is quite likely that Factor H is displaced from the borrelial outer surface, leaving the cells unprotected to the detrimental effects of complement [77,78]. To what extent Factor H is able to protect *Borreliae* from human complement by binding to Erp proteins during certain stages of infection remains to be determined.

Binding of murine Factor H to Erp proteins has been reported by different investigators employing various approaches for detecting protein-protein interactions [69,70,71,100], while others were unable to confirm binding of murine Factor H to Erp orthologs, thereby leading to the assumption that mice *per se* are probably not an appropriate model to study Factor H-mediated immune evasion [72]. Furthermore, these authors showed that ErpA and ErpP differ in their ability to bind Factor H from different animals (Table 3), thus suggesting that sequence variations among Erp proteins are responsible for the species-specific Factor H binding pattern [72]. Additional Factor H-binding proteins, potentially belonging to the Erp protein family, due to the similar molecular weights, have been identified among *B. afzelii*, *B. garinii*, *B. bavariensis*, *B. valaisiana*, and *B. andersonii*. These were shown to bind to Factor H derived from mice, rats, dogs, or cats [70]. Comparative sequence analysis of a range of *B. burgdorferi* strains revealed multiple Erp proteins exhibiting a larger or smaller degree of amino acid sequence identity, which can be simultaneously produced by individual bacteria. These proteins are believed to possess different functions and could in theory contribute to the pathogenesis of Lyme disease spirochetes (e.g., by binding to Factor H of distinct animal species), as has been shown for ErpG, ErpX, and ErpY [74]. The considerable sequence variation and the restricted species-specific binding pattern of Factor H may constitute further evidence for a role of this complement regulator in immune evasion of spirochetes in different reservoir hosts. Pursuing the concept of Factor H-mediated immune evasion as an important factor in spirochete ecology, it should follow that the Factor H binding pattern should match the serum resistance phenotype of the respective genospecies. As depicted in Table 4, there is indeed a striking correlation between Factor H-binding and serum resistance for most borrelial species and sera analyzed, except for *B. bavariensis* in human, rat, cat, and dog serum, *B. afzelii* in rat serum, *B. valaisiana* and *B. japonica* in cat serum, as well as *B. bissettii* in dog serum. Concerning *B. bavariensis*, it has recently been shown that two CspA orthologs, BGA66 and BGA71, inhibit complement activation of human serum independently of binding of Factor H by direct interaction with the terminal pathway, resulting in inhibition of TCC assembly [68]. As the inhibitory capacity of BGA66 and BGA71 on animal sera has not been investigated yet, it is impossible to make a final conclusion concerning host specificity drawn from the data available. Of note, it is well-known that spirochetes tend to lose plasmids during continuous cultivation, thus depending on the genetic composition, the borrelial strains used in the different studies may or may not produce various Factor H-binding proteins. This might also be an explanation for the discrepancies observed between different studies. As expected, a strict correlation could not be found for borrelial strains displaying an intermediate serum-resistant phenotype in the corresponding sera. More importantly, the inability to bind to Factor H correlated well with a serum susceptible phenotype (Table 4), underlining the importance of Factor H-dependent complement evasion by *Borreliae* in their diverse hosts.

## 6. Interaction of *Borreliae* with Additional Complement Proteins

Binding of additional serum factors, in particular complement regulatory proteins, may also have an impact in facilitating serum resistance of Lyme disease spirochetes. It has recently been shown that *B. burgdorferi* is able to directly interact with the C1 complex through the outer surface protein BBK32. Additionally, *Borreliae* can interact with components of the TCC through CspA, resulting in an efficient termination of the classical and terminal pathway, thus protecting spirochetes from being killed by human complement [90,101]. Concerning C1-INH, which is known to bind to relapsing fever spirochetes [102], there is no evidence of an interaction of Lyme disease spirochetes with this particular regulator of the classical and lectin pathway [67,68,103]. Binding of native C4BP, the key complement regulator of the classical pathway, has been reported for *B. burgdorferi* and *B. garinii* [104]. Furthermore, recombinant human C4BP has been shown to bind to *B. burgdorferi*, *B. afzelii*, *B. garinii*, *B. bavariensis*, and, to a somewhat weaker extent, to *B. valaisiana*, *B. lusitaniae*, *B. bissettii*, and *B. japonica* using a Far-Western blot approach [103]. The same authors also observed binding of recombinantly produced ovine C4BP to *B. burgdorferi*, while none of the tested borrelial strains bound to recombinantly produced bovine C4BP. Further analyses revealed that several borrelial species, including *B. afzelii*, *B. garinii*, *B. bavariensis*, and *B. andersonii*, appear to interact with recombinantly produced human vitronectin, a specific inhibitor of the terminal pathway, but not with vitronectin of ovine or bovine origin [103]. Pull down assays using recombinant complement regulators following mass spectrometry led to the identification of several proteins interacting with human Factor H (CspA orthologs of *B. burgdorferi* and *B. afzelii*, BG0407 of *B. bavariensis*), C4BP (hypothetical outer surface proteins of *B. afzelii*, *B. burgdorferi* and *B. garinii*, OspA of *B. burgdorferi*), and vitronectin (variable large protein of *B. garinii*) [103]. Future studies are necessary to confirm binding of these complement regulators to the respective borrelial proteins, using native complement proteins in order to determine the biological relevance of the observed protein-protein interaction in the context of the well-known resistance patterns of the respective borrelial species.

## 7. Future Perspectives

Over the past 15 years, numerous proteins of Lyme disease spirochetes have been described that interact specifically with human complement whereby a few studies also focused on the identification of proteins interacting with serum components of various animals [11,47,70,83,101]. There is a growing body of evidence suggesting an important role for the innate immune system of a particular host in determining the transmissibility and the overall dispersal of spirochetes. However, only fragmentary information is available on specific borrelial-derived determinants that are crucial for evading complement as the first line of host defense. 

Comprehensive proteomics analyses, including expression and interaction proteomics, may foster the identification of potential complement-interacting proteins of spirochetes as well as other important veterinary pathogens (*Babesia* spp., *Streptococcus equi*, *Mycobacterium* spp., *Rickettsia* spp.). Of note, whenever practical, a thorough biochemical analysis should be conducted to further characterize and quantify protein-protein interactions by different approaches (e.g., ELISA, isothermal calorimetry, surface-plasmon resonance, or microscale thermophoresis, whereby the latter grants measurement of biomolecular interaction under immobilization-free, close-to-native conditions) [105]. Furthermore, one should always bear in mind that the collected data have to be carefully interpreted in the light of a rational syllogism.

## 8. Conclusions 

Lyme disease spirochetes have developed an array of sophisticated strategies to establish infection and to circumvent host innate immunity by fending off host complement attack through acquisition of soluble complement regulators of different vertebrate hosts. In particular, the species-specific binding patterns of Factor H, in the majority of cases, matches the serum resistance phenotype of the respective borrelial genospecies, thereby suggesting that this molecular interaction plays an important role in immune evasion of Lyme disease spirochetes during the transmission cycles of various vertebrate hosts. Moreover, *Borreliae* exploit further strategies, namely the direct interaction with complement components to manipulate, influence, and terminate complement activation. As this scenario has only been described for human complement, similar interaction profiles can be expected for animal-derived components. Clearly, animal complement regulators are able to bind to different borrelial proteins, and further studies are required to validate the biological relevance of the interactions *in vitro* as well as *in vivo*. However, the hypothesis of a complement-mediated selection determining the host range of a particular borrelial species presented here requires reconciliation with the conflicting observations described. In closing, further investigations will undoubtedly contribute to an increased understanding of how *Borreliae* persistently infect their hosts and which specific determinants are involved and responsible for survival and persistence. 

## Figures and Tables

**Table 1:** Serum susceptibility pattern of different borrelial species to human and animal sera.

Species ^a^	*B. burgdorferi*	*B. afzelii*	*B. bavariensis*	*B. garinii*	*B. valaisiana*	*B. lusitaniae*	*B. japonica*	*B. bissettii*	*B. andersonii*
Human	**R**	**R**	**R**	**S**	**S**	**S**	**R**	**I**	**S**
Mouse	**R**	**R**	**R**	**S**	**S**	**ND**	**R**	**R**	**ND**
Rat	**S**	**R**	**R**	**S**	**ND**	**ND**	**ND**	**ND**	**ND**
Hamster	**R**	**R**	**R**	**S**	**S**	**S**	**R**	**ND**	**ND**
Squirrel	**R**	**R**	**R**	**S**	**S**	**ND**	**R**	**ND**	**ND**
Cat	**I**	**R**	**R**	**I**	**R**	**ND**	**R**	**ND**	**ND**
Dog	**I**	**R**	**R**	**S/I**	**I**	**S**	**I**	**R**	**R**
Mouflon	**I**	**R**	**R**	**R/I**	**R**	**R**	**R**	**R**	**I**
Lynx	**I**	**I**	**R**	**I**	**R**	**S**	**S**	**R**	**I**
Wolf	**I**	**S**	**R**	**S/I**	**S**	**S**	**S**	**R**	**I**
Sheep	**I**	**S**	**S**	**S**	**S**	**R**	**R**	**S/R**	**I**
Horse	**I**	**S**	**S**	**S**	**S**	**S**	**S**	**ND**	**ND**
Pig	**I**	**S**	**S**	**S**	**S**	**S**	**S**	**ND**	**ND**
Rabbit	**I**	**S**	**ND**	**S**	**ND**	**ND**	**ND**	**I**	**ND**
Pheasant	**I**	**S**	**S**	**R**	**R**	**S**	**S**	**ND**	**ND**
Blackbird	**I**	**S**	**S**	**R**	**R**	**S**	**S**	**ND**	**ND**
Goat	**S**	**S**	**ND**	**S**	**ND**	**ND**	**ND**	**ND**	**ND**
Bovine	**S**	**S**	**S**	**S**	**S**	**S**	**S**	**S**	**S**
Deer	**S**	**S**	**S**	**S**	**S**	**S**	**S**	**S**	**S**
Eur. Bison	**S**	**S**	**S**	**S**	**S**	**S**	**S**	**S**	**S**
Lizard	**S**	**S**	**S**	**R**	**R**	**R**	**S**	**S**	**ND**
Quail	**R**	**ND**	**ND**	**ND**	**ND**	**ND**	**ND**	**S**	**ND**

^a^ variations in the serum susceptibility patterns have been reported for the polymorphic genospecies *B. garinii* [46]. *B. spielmanii* has not been included due to the lack of available data but this genospecies resists complement-mediated killing by human serum [65]; Light blue, serum-resistant (R); grey, intermediate serum-resistant (I); yellow, serum-sensitive (S), transparent, no data available (ND); orange, phenotype unclear.

**Table 2:** Binding of human and animal factor H to different borrelial species.

Species	*B. burgdorferi*	*B. afzelii*	*B. bavariensis*	*B. garinii*	*B. valaisiana*	*B. lusitaniae*	*B. japonica*	*B. bissettii*	*B. andersonii*
Human	+	+	−	−	−	−	(+)	(+)	+
Mouse	(+)	+	(+)	−	−	−	+	+	+
Rat	−	−	−	−	−	−	+	−	−
Cat	−	+	−	−	−	−	+	−	−
Dog	−	−	−	−	+	−	+	−	−
Sheep	+	−	−	−	−	−	−	+	−
Horse	(+)	−	−	−	−	−	−	−	−
Cattle	−	−	−	−	−	−	−	−	−

Symbols in parenthesis indicate controversial results of Factor H binding to the respective borrelial species. In each case, further studies are required to unambiguously determine whether those species indeed interact with factor H.

**Table 3:** Binding of human and animal factor H to CRASPs of *B. burgdorferi* s.s.

Serum Source ^a^	CspA_Bb_ (BBA68)	CspA_Ba_	CspZ (BBH06)	ErpP (BBN38)	ErpC	ErpA (BBL39)
Human	+	+	+	+	+	+
Mouse	(+)	(+)	+	(+)	(+)	(+)
Rat	−	−	−	−	+	−
Rabbit	−	ND	+/−	−	ND	−
Cat	−	−	−	−	ND	−
Dog	−	−	−	+	ND	−
Sheep	−	ND	ND	−	ND	−
Horse	−	−	−	−	ND	−
Cow	−	−	+	+	ND	−
Pig	−	ND	+	+	ND	−

Symbols in parenthesis indicate controversial results of Factor H binding to the respective CRASP protein. Further studies are required to unambiguously determine whether the respective proteins interact with factor H; **^a^** binding of human and animal factor H to purified proteins was assessed by different Western blotting approaches [70,72,75,77,78,88,89,95]; ND, no data available; Bb, *B. burgdorferi* s.s.; Ba, *B. afzelii*; Csp, complement regulator-acquiring surface protein; Erp, OspE/F-related protein; synonyms of protein names are indicated in brackets.

**Table 4:**
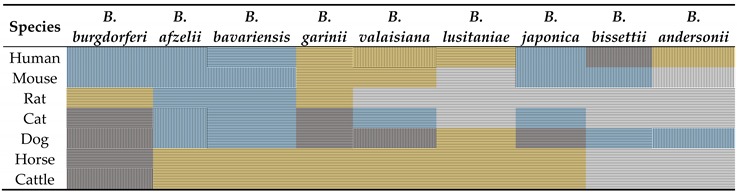
Correlation of serum susceptibility and Factor H binding for different borrelial species.

Light blue, serum-resistant; grey, intermediate serum-resistant; yellow, serum-sensitive; transparent, no data for the serum susceptibility phenotype are available; vertical stripes, binding of factor H, horizontal stripes, no binding of factor H.
